# Short-term longitudinal clinical, biochemical, and quality of life outcomes of medical or surgical therapy in unilateral primary aldosteronism

**DOI:** 10.3389/fendo.2025.1558837

**Published:** 2025-08-29

**Authors:** Yuichi Yoshida, Kentaro Sada, Yoshiro Matsuo, Satoshi Nagai, Naoki Matsuda, Takaaki Noguchi, Chiaki Yonezu, Nao Imaishi, Machiko Morita, Yumi Mori, Shotaro Miyamoto, Yoshinori Ozeki, Koro Gotoh, Takayuki Masaki, Hirotaka Shibata

**Affiliations:** ^1^ Department of Endocrinology, Metabolism, Rheumatology and Nephrology, Faculty of Medicine, Oita University, Yufu, Japan; ^2^ Faculty of Medicine, Oita University, Yufu, Japan; ^3^ Faculty of Welfare and Health Sciences, Oita University, Oita, Japan; ^4^ Geriatric Nursing, Department of Nursing, Faculty of Medicine, Oita University, Yufu, Japan

**Keywords:** adrenalectomy, hypertension, mineralocorticoid receptor antagonist, primary aldosteronism, quality of life

## Abstract

**Context:**

Unilateral primary aldosteronism (uPA) is a surgically curable form of hypertension, frequently associated with resistant hypertension and cerebro-cardiovascular complications. Primary aldosteronism (PA) also negatively affects quality of life (QOL).

**Objective:**

This study sought to compare the efficacy of mineralocorticoid receptor antagonists (MRAs) and adrenalectomy (ADX) in the treatment of patients with uPA over time within the same patients and to evaluate the efficacy of MRAs in patients with bilateral PA (bPA).

**Methods:**

Subtype diagnosis of PA was based on adrenal vein sampling results. Clinical parameters, including blood pressure, serum potassium (K), active renin concentration (ARC), aldosterone levels, estimated glomerular filtration rate, and Medical Outcomes Study 36-Item Short-Form Health Survey (SF-36) scores, were investigated before and after MRA treatment in all PA patients, as well as after ADX in patients with uPA.

**Results:**

The study included 56 patients with bPA and 20 patients with uPA. Changes in parameters with MRA treatment were similar between patients with and without uPA, except for a greater increase in K in patients with uPA. Among patients with uPA, systolic blood pressure and K improved with MRA treatment and showed further improvement following ADX. Diastolic blood pressure and ARC also improved following MRA treatment, with no significant differences observed compared to ADX. SF-36 scores showed no improvement with MRA treatment, but significantly improved after ADX.

**Conclusion:**

The findings showed that ADX generally provides superior clinical, biochemical, and QOL outcomes compared to MRAs in patients with uPA.

## Introduction

Primary aldosteronism (PA) accounts for 5–13% of hypertension cases (1–4) and is a common cause of secondary hypertension. PA is categorized into two major subtypes: unilateral primary aldosteronism (uPA), which is typically caused by an aldosterone-producing adenoma (APA), and bilateral primary aldosteronism (bPA) or idiopathic hyperaldosteronism (IHA), which is generally caused by bilateral adrenal hyperplasia (BAH) (3, 5, 6). Patients diagnosed with uPA often exhibit markedly elevated plasma aldosterone levels, spontaneous hypokalemia, and lower plasma renin activity (PRA) or concentrations compared to those with bPA. The former subtype is frequently associated with resistant hypertension and cerebro-cardiovascular complications (7, 8). Additionally, numerous studies have demonstrated that PA adversely affects quality of life (QOL) (9–18).

Most clinical practice guidelines recommend unilateral adrenalectomy (ADX) for the treatment of patients with uPA, and mineralocorticoid receptor antagonists (MRAs) for those with bPA (3, 5, 6). Although several studies have attempted to compare the efficacy of MRAs and ADX for uPA treatment (19–24), these studies were not randomized controlled trials, and the rationale behind treatment choices was often insufficiently explained. Moreover, no prior study has assessed the impact of these treatments on QOL. Zhang et al. (25) presented data comparing perioperative blood pressure, serum potassium (K), and the incidence of hyperkalemia following ADX, but their study did not directly compare MRA and ADX treatments. The design of this study is similar to that employed by Kisimoto et al. (26) except that this study evaluated QOL or the extent to which the effects of MRAs differ between patients with uPA and those with bPA, which were not addressed in their study. Other studies have evaluated the impact of ADX and MRAs on QOL but these studies have typically focused on patients with uPA and bPA separately (14, 27).

The purpose of this study was to evaluate the differences in treatment efficacy between MRA and ADX for uPA using clinical and biochemical data in conjunction with QOL assessments. Specifically, we compared the effects of preoperative MRA treatment and subsequent ADX treatment in patients with uPA over time, as well as the effects of MRA treatment in patients with bPA. In addition, to assess the impact of MRA treatment in patients with uPA, we examined its effects relative to those observed in patients with bPA.

## Materials and methods

### Study design

**Figure 1:**
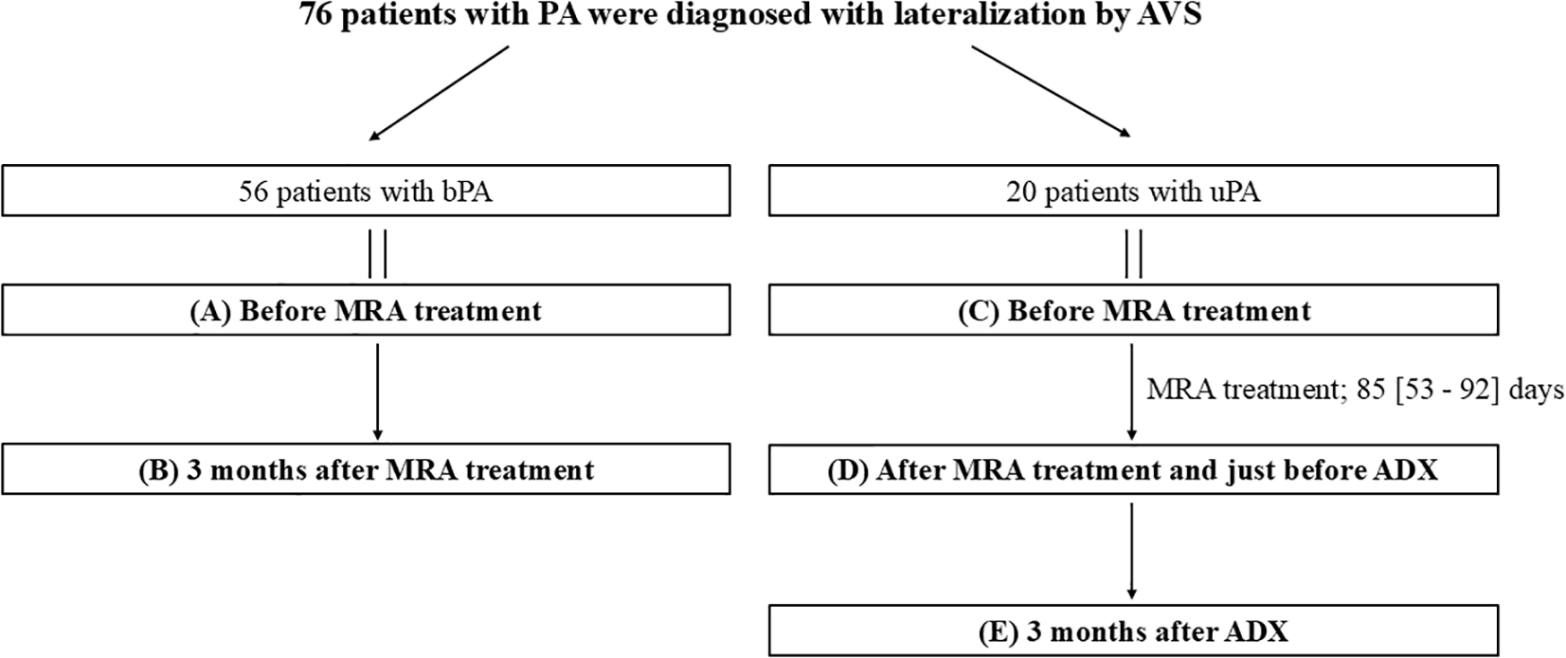
Overview of the study. Data are presented as medians [first quartile–third quartile]. ADX, adrenalectomy; bPA, bilateral primary aldosteronism; MRA, mineralocorticoid receptor antagonist; PA, primary aldosteronism; uPA, unilateral primary aldosteronism.

This observational study was conducted at Oita University Hospital between July 2017 and March 2023. Of 165 patients diagnosed with PA, 56 patients were identified as patients with bPA based on AVS results. A total of 32 patients were confirmed to have uPA through AVS. Of these, all patients with bPA and 20 patients with uPA who had QOL data available both before and after treatment were included in the study. Patients with uPA were not receiving MRAs at the time of diagnosis, were compliant with MRA treatment following their uPA diagnosis, and were available for parameter evaluation both prior to and 3 months after ADX. Patients with bPA initiated MRA treatment after diagnosis, and treatment effects were assessed 3 months later. In patients with uPA, MRAs were administered for a short-term period prior to ADX to stabilize blood pressure and serum K levels. In contrast, in patients with bPA, MRAs were administered with the aim of achieving long-term and sustained stabilization of blood pressure and biochemical parameters. The parameters evaluated included systolic blood pressure (SBP), diastolic blood pressure (DBP), serum K levels, estimated glomerular filtration rate (eGFR), serum aldosterone concentration (SAC), active renin concentration (ARC), aldosterone-renin ratio (ARR), and QOL, which was measured using the Medical Outcomes Study 36-Item Short-Form Health Survey (SF-36) (**Figure 1**).

### Laboratory tests

ARC was determined using the Accuraseed Renin Kit, which utilizes the Chemiluminescent Enzyme Immunoassay (CLEIA) method (FUJIFILM Wako Pure Chemical Corp., Osaka, Japan). SACs were also assessed using the CLEIA method. For measurements conducted before July 2021, the Accuraseed Aldosterone Kit (Fujifilm Wako Pure Chemical Corp.) was used. Measurements conducted after August 2021 were performed using the Accuraseed Aldosterone S Kit (Fujifilm Wako Pure Chemical Corp.). Since the SACs obtained with the Accuraseed Aldosterone Kit are consistent with those obtained by RIA, and the SACs obtained with the Accuraseed Aldosterone S Kit correspond to those measured by liquid chromatography-tandem mass spectrometry (LC-MS/MS), aldosterone values were adjusted using the following formula (28):

SAC (equivalent to LC-MS/MS method) = 0.765 × SAC (equivalent to RIA method) - 33.7.

The method used to measure urinary aldosterone in this study changed over time. Specifically, until March 2020, measurements were conducted using the Spac-S Aldosterone kit (Fujirebio Inc., Tokyo, Japan) based on the RIA method. From April 2020 to March 2022, the Determinar CL Aldosterone kit (Minaris Medical Co., Ltd., Mountain View, CA, USA) was used, utilizing a competitive CLEIA method that is comparable to the RIA method. Since April 2022, the Lumipulse Presto Aldosterone kit (Fujirebio Inc.) has been used, employing a sandwich CLEIA method that is consistent with the LC-MS/MS method. This new sandwich CLEIA method has been reported to have lower values than the conventional CLEIA method and the RIA method (28, 29), and the diagnostic cutoff value is set lower in PA in accordance with treatment guidelines in Japan (6) compared to guidelines in other countries.

### Diagnosis of PA

Patients were diagnosed with PA in accordance with treatment guidelines in Japan (6). Screening tests involved morning measurements of ARC and SAC, with a positive result defined as a SAC/ARC (pg/mL) ratio > 40 and a provisionally positive result as a ratio of > 20. Confirmatory tests, including the saline infusion test (SIT), captopril challenge test (CCT), and oral salt loading test (OSLT), were subsequently performed. For SIT, 2 L of saline was administered intravenously over 4 h while the patient remained in a supine position. A test result was considered positive if SAC was > 60 pg/mL and provisionally positive if SAC was > 12 pg/mL. CCT involved the administration of 50 mg of captopril, followed by blood collection 90 min later. The test was considered positive when the SAC/ARC ratio was > 40 and provisionally positive when it was > 20. For OSLT, a 24-h urine sample was collected after the patient consumed a high-salt diet. Prior to March 2022, a urinary aldosterone level > 8 µg/day was considered positive if urinary sodium excretion was > 170 mEq/day. Starting in April 2022, following a change in the measurement kit, a 24-h urinary aldosterone level > 6 µg/day was considered positive.

### Adrenal vein sampling

AVS was employed to localize the source of PA, and all patients diagnosed with uPA in this study underwent AVS. Localization was determined in accordance with the guidelines established by the Japan Endocrine Society (6). Following the administration of adrenocorticotropic hormone (ACTH), successful catheter placement was confirmed if the adrenal venous cortisol level was at least five times higher than the cortisol concentration in peripheral vessels. A diagnosis of unilateral PA was made when the lateralized ratio (LR) was ≥ 4 and the contralateral ratio (CR) was < 1.

### MRAs

MRAs were used from the time of PA diagnosis until ADX for uPA or as bPA therapy. The MRAs of choice were spironolactone, eplerenone, or esaxerenone. Esaxerenone is a new MRA with proven efficacy in PA (30) and is currently widely used in Japan (18).

### ADX

All ADX procedures were performed laparoscopically by urologists at Oita University Hospital. MRAs were administered until the day prior to ADX, but were discontinued post-ADX.

### Blood pressure measurement

SBP and DBP were measured at multiple time points: before MRA treatment, preoperatively after hospital admission, and following a period of bed rest. Blood pressure at 3 months post-ADX was determined as the average of three home measurements.

### QOL assessment

QOL was assessed using the SF-36 questionnaire, a widely used and validated tool for evaluating health-related QOL in the Japanese population (31–33). Normative data from 2,279 healthy Japanese individuals were used as a reference for comparison with the study participants. For statistical comparisons, the mean ± 1 standard deviation SF-36 score for the healthy cohort was adjusted/standardized to 50 ± 10 points. The SF-36 questionnaire comprises eight subscales: Physical Functioning (PF), Role-Physical (RP), Bodily Pain (BP), General Health (GH), Vitality (VT), Social Functioning (SF), Role-Emotional (RE), and Mental Health (MH).

### Statistical analysis

Data are expressed as median [first quartile–third quartile], except for SF-36 scores, which are presented as mean ± standard deviation. Statistical significance was defined as P < 0.05. For nonparametric data, including K, eGFR, SAC, ARC, and ARR, the Mann-Whitney *U* test was used for comparisons between two groups, while the Friedman test with Dunn’s multiple comparison test was used for comparisons across three groups. For parametric data, paired and unpaired *t*-tests were used for two-group comparisons, and repeated-measures one-way analysis of variance (ANOVA) was used to compare three groups over time, followed by Tukey’s multiple comparison test. The Chi-square test was performed to compare the gender ratio and the rate of achieving ARC ≥5 pg/mL. All statistical analyses were conducted using GraphPad Prism (version 9, GraphPad Software, Boston, MA, USA).

### Ethical considerations

This study was approved by the Oita University Ethics Committee (Approval Nos. 909 and 1761) and was conducted in accordance with the principles outlined in the Declaration of Helsinki. Written informed consent was obtained from each patient prior to enrollment in the study.

## Results

### Patient background

**Figure 2:**
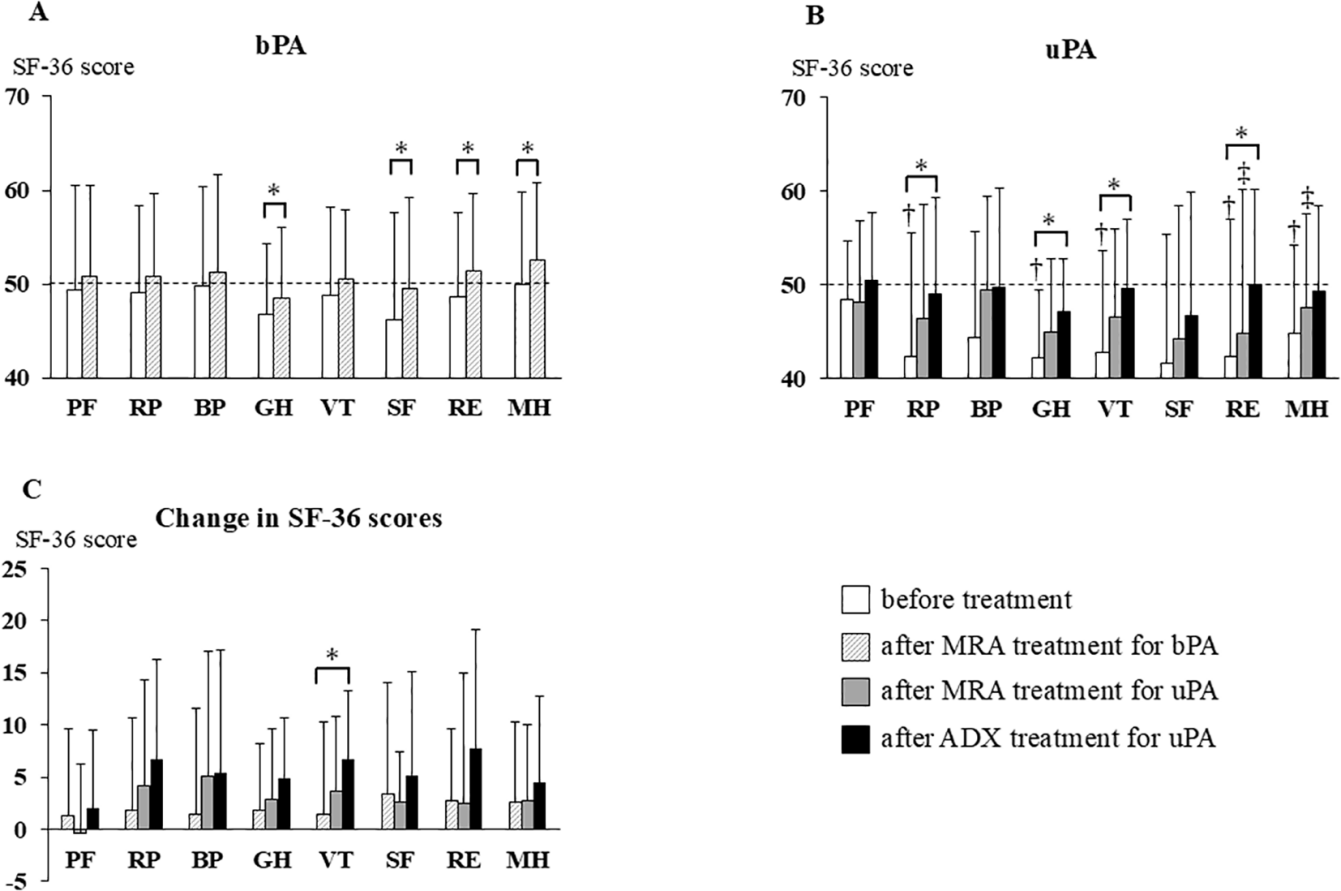
Changes in SF-36 scores before treatment, after MRA, and after ADX. Bars represent means + standard deviations. The mean SF-36 score for the Japanese general population was standardized to 50 points (dotted line) with a standard deviation of 10 points. **(A)** SF-36 scores before and after MRA treatment in patients with bPA; **(B)** SF-36 scores before treatment, after MRA treatment, and after ADX in patients with uPA; **(C)** Changes in SF-36 scores after MRA treatment in patients with bPA and after MRA treatment and ADX in patients with uPA. *P <0.05; †, *P* < 0.05 vs patients with bPA before treatment; ‡, *P* < 0.05 vs patients with bPA after MRA treatment. ADX, adrenalectomy; bPA, bilateral primary aldosteronism; MRA, mineralocorticoid receptor antagonist; SF-36, Medical Outcomes Study 36-Item Short-Form Health Survey; uPA, unilateral primary aldosteronism; PF, Physical Functioning; RP, Role-Physical; BP, Bodily Pain; GH, General Health; VT, Vitality; SF, Social Functioning; RE, Role-Emotional; MH, Mental Health.

Patients with bPA began MRA treatment with one of the following: spironolactone, eplerenone, or esaxerenone. Patients with uPA underwent MRA treatment following their diagnosis and proceeded to ADX after a median time of 85 days (interquartile range [IQR]: 53–92 days) (**Figure 1**). Among the patients with uPA, 9 were treated with spironolactone (the dose of 25 mg) and the remaining 11 with esaxerenone (the dose of 2.5 and 5 mg in 8 and 3 patients, respectively). Among patients with bPA, 8 were treated with spironolactone (the dose of 25 and 50 mg in 7 and 1 patients, respectively), 19 with eplerenone (the dose of 25, 50, and 100 mg in 9, 9, and 1 patients, respectively), and 29 with esaxerenone (the dose of 2.5, and 5 mg in 27 and 9 patients, respectively). The dosage of MRAs was adjusted based on clinical and biochemical parameters including BP, K, and ARC. Among patients with uPA, 5 (25.0%) achieved an ARC ≥5 pg/mL after MRA treatment, and 7 (35.0%) achieved this level after ADX. Among patients with bPA, 26 (46.4%) achieved an ARC ≥5 pg/mL. None of these results were statistically significant. Throughout the observational period, no cerebro-cardiovascular events, MRA-related side effects, or perioperative complications from ADX were reported. Comparing patients with uPA and bPA before MRA treatment (**Figure 1A** vs **1C**), patients with uPA had lower K and ARC values, and higher SAC and ARR values than patients with bPA (**Table 1**). For the SF-36 questionnaire, patients with uPA had significantly lower scores than patients with bPA in RP, GH, VT, RE, and MH (RP, 49.0 ± 9.3 vs 42.3 ± 13.2; GH, 46.8 ± 7.6 vs 42.1 ± 7.3; VT, 46.2 ± 11.5 vs 41.6 ± 13.8; RE, 48.6 ± 9.0 vs 42.3 ± 14.6; and MH, 50.0 ± 9.8 vs 44.8 ± 9.4, respectively; *P <*0.05). No differences were observed in PF, BP, SF (PF, 49.4 ± 11.1 vs 48.5 ± 6.1; BP, 49.8 ± 10.6 vs 44.4 ± 11.2; and SF, 48.8 ± 9.4 vs 42.8 ± 10.9, respectively) (**Figure 2B**).

**Table 1:** Parameters before and after treatment in patients with bPA and uPA.

	bPA				uPA					
	Before treatment		After MRAs		Before treatment		After MRAs		3 months after ADX	
N (male/female)	56	(24/32)			20	(10/10)				
age	54	[46 - 63]			53	[43 - 61]				
BMI (kg/m^2^)	24.1	[21.3 - 26.6]			23.3	[22.2 - 27.9]				
SBP (mmHg)	139	[127 - 150]	126	[120 - 135]^a^	141	[129 - 152]	128	[119 - 137]^b^	121	[110 - 130]^b,c,d^
DBP (mmHg)	83	[76 - 94]	80	[72 - 88]^a^	92	[81 - 96]	83	[74 - 86]^b^	79	[75 - 85]^b^
K (mmol/L)	3.9	[3.7 - 4.1]	4.3	[4.1 - 4.5]^a^	3.0	[2.8 - 3.5]^a^	3.9	[3.7 - 4.3]^b,d^	4.4	[4.2 - 4.9]^b,c^
eGFR (mL/min/1.73m^2^)	75.9	[69.1 – 88.6]	68.8	[62.6 – 78.7]^a^	78.8	[66.4 - 90.1]	62.5	[53.3 - 76.2]^b^	56.7	[47.6 - 70.1]^b,c,d^
ARC (pg/mL)	2.4	[1.0 – 4.0]	4.4	[2.2 – 10.4]^a^	1.3	[0.7 - 3.3]^a^	2.5	[1.5 - 5.2]^b,d^	3.9	[2.5 - 6.8]^b^
Post-treatment ARC ≥5 achievement rate (%)			46.4				25.0		35.0	
SAC (pg/mL)	114	[78 - 186]	159	[109 - 200]^a^	335	[176 - 412]^a^	218	[151 - 331]^d^	22	[7 - 64]^b,c,d^
ARR	49	[28 - 92]	28	[15 - 61]^a^	244	[78 - 404]^a^	87	[32 - 214]^d^	8	[3 - 15]^b,c,d^
antihypertensive agents (N)										
MRAs	0		56		0		20		0	
spironolactone	0		8		0		9		0	
eplerenone	0		19		0		0		0	
esaxerenone	0		6		0		11		0	
CCBs	43		39		17		15		10	
ARBs	3		2		2		2		1	
diuretics	1		1		0		0		0	
α-blockers	2		2		5		3		0	
β-blockers	2		2		2		2		1	
potassium supplements (N)	0		0		12		7		0	

Data are presented as medians [first quartile–third quartile]. ADX, adrenalectomy; ARB, angiotensin II receptor blocker; ARC, active renin concentration; ARR, aldosterone–renin ratio; BMI, body mass index; CCB, calcium channel blocker; DBP, diastolic blood pressure; eGFR, estimated glomerular filtration rate; K, serum potassium; MRA, mineralocorticoid receptor antagonist; SAC, serum aldosterone concentration; SBP, systolic blood pressure; uPA, unilateral primary aldosteronism; α-blocker, alpha-adrenergic blocker; β-blocker, beta-adrenergic blocker.

a *P <*0.05 vs. before treatment in bPA.

b *P <*0.05 vs. before treatment in uPA.

c *P <*0.05 vs. after MRAs in uPA.

d *P <*0.05 vs. after MRAs in bPA.

### Comparison of clinical and biochemical data after MRA treatment between patients with bPA and uPA

After MRA treatment (**Figures 1B** vs **1D**), patients with uPA had lower K and ARC values, and higher SAC and ARR values than patients with bPA. These findings were consistent with the results obtained before MRA treatment (**Table 1**). On the SF36 questionnaire, no differences were observed in PF, RP, BP, GH, VT or SF domain scores between patients with uPA and bPA (PF, 50.8 ± 9.7 vs 48.1 ± 8.7; RP, 50.8 ± 8.8 vs 46.4 ± 12.1; BP, 51.2 ± 10.5 vs 49.5 ± 9.9; GH, 48.5 ± 7.4 vs 45.0 ± 7.8; VT, 50.5 ± 7.4 vs 46.5 ± 9.4; and SF, 49.5 ± 9.7 vs 44.1 ± 14.3, respectively). However, scores for the RE and MH domains remained significantly lower in patients with uPA (RE, 51.3 ± 8.3 vs 44.8 ± 15.3; and MH, 52.6 ± 8.2 vs 47.5 ± 9.9, respectively; *P <*0.05) (hatched bars in **Figure 2A** and shaded bars in **Figure 2B**).

### Comparison of clinical and biochemical data between patients with bPA after MRA treatment and patients with uPA after ADX

When comparing patients with bPA after MRA treatment to patients with uPA after ADX (**Figures 1B** vs **1E**), patients with uPA exhibited lower SBP, eGFR, SAC and ARR than patients with bPA (**Table 1**). On the SF36 questionnaire, differences in all domain scores disappeared between patients with bPA after MRA treatment and patients with uPA after ADX (PF, 50.8 ± 9.7 vs 50.4 ± 7.3; RP, 50.8 ± 8.8 vs 48.9 ± 10.3; BP, 51.2 ± 10.5 vs 49.8 ± 10.6; GH, 48.5 ± 7.4 vs 47.0 ± 5.8; VT, 50.5 ± 7.4 vs 49.5 ± 7.4; SF, 49.5 ± 9.7 vs 46.7 ± 13.1; RE, 51.3 ± 8.3 vs 50.0 ± 10.1; and MH, 52.6 ± 8.2 vs 49.3 ± 9.1, respectively) (**Figures 2A, B**).

### Clinical and biochemical data after MRA treatment in patients with bPA

Comparing the data before and after MRA treatment in patients with bPA (**Figures 1A** vs **1B**), significant reductions were observed in SBP, DBP, eGFR, and ARR. Conversely, K, ARC, and SAC were significantly higher after MRA treatment compared to before treatment (**Table 1**). In addition, on the SF-36 scores, GH, SF, RE, and MH showed significant improvement after MRA treatment (GH, 46.8 ± 7.6 vs 48.5 ± 7.4; SF, 46.2 ± 11.5 vs 49.5 ± 9.7; RE, 48.6 ± 9.0 vs 51.3 ± 8.3; and MH, 50.0 ± 9.8 vs 52.6 ± 8.2, respectively; *P <*0.05) (**Figure 2A**).

### Clinical and biochemical data after MRA treatment and ADX in patients with uPA

In patients with uPA, comparisons of data before and after MRA treatment and after ADX (**Figures 1C** vs **1D** vs **1E**) revealed that SBP and eGFR were reduced after both MRA treatment and ADX, with further reductions observed after ADX. DBP also decreased following both treatments but did not differ significantly between MRA and ADX. Compared to baseline levels, K levels increased following both treatments, with a greater increase observed post-ADX. ARC increased after both treatments but showed no significant differences between MRA and ADX. SAC remained unchanged with MRA treatment but decreased significantly after ADX. ARR showed no significant change after MRA treatment but was significantly reduced after ADX. Additionally, the number of antihypertensive medications required was notably reduced after ADX (**Table 1**).

On the SF-36 questionnaire, compared with before treatment, MRA treatment did not significantly improve SF-36 scores, whereas ADX led to marked improvements in the RP, GH, VT, and RE domains (RP, 42.3 ± 13.2 vs 48.9 ± 10.3; GH, 42.1 ± 7.3 vs 47.0 ± 5.8; VT, 41.6 ± 13.8 vs 46.7 ± 13.1; and RE, 42.3 ± 14.6 vs 50.0 ± 10.1, respectively; *P <*0.05) (**Figure 2B**).

### Comparison of changes in clinical and biochemical data and SF-36 scores after each treatment (MRA treatment in patients with uPA and bPA and ADX treatment in patients with uPA)

**Figure 3:**
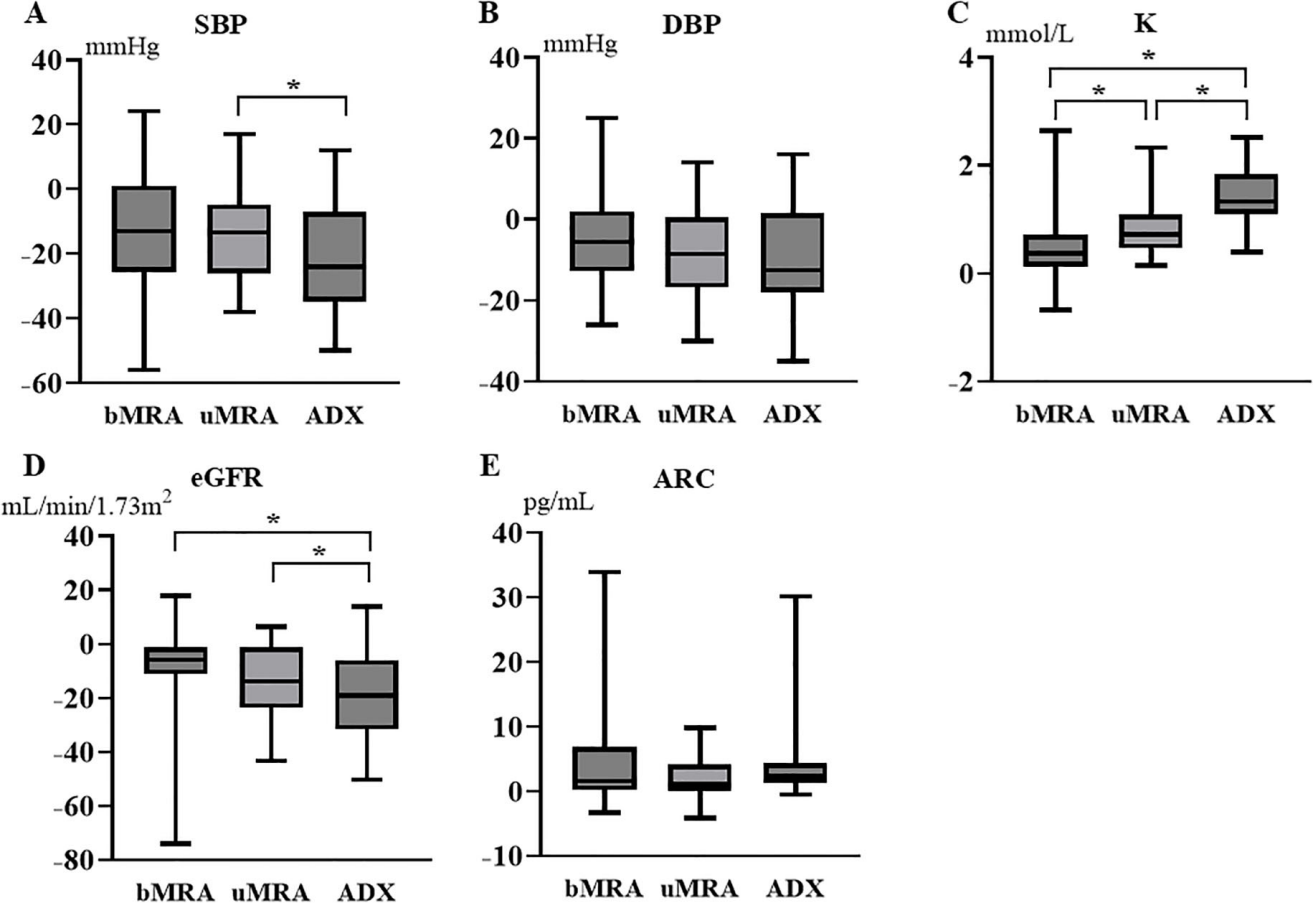
Changes in SBP, DBP, K, eGFR, and ARC after MRA and ADX compared to before treatment. The box plot shows the median value (horizontal bar inside the box), the first quartile–third quartile (box height), and the minimum–maximum range (vertical bars). **(A)** Changes in SBP; **(B)** Changes in DBP; **(C)** Changes in K; **(D)** Changes in eGFR; **(E)** Changes in ARC. *P < 0.05. ADX, adrenalectomy; ARC, active renin concentration; bMRA, MRA treatment in bilateral primary aldosteronism; DBP, diastolic blood pressure; eGFR, estimated glomerular filtration rate; K, serum potassium; MRA, mineralocorticoid receptor antagonist; SBP, systolic blood pressure; uMRA, MRA treatment in unilateral primary aldosteronism.

As described above, patients with uPA had more severe clinical parameters than patients with bPA before the initiation of MRA treatment, making direct comparisons of post-treatment values alone insufficient to calculate treatment effects. Therefore, we compared the treatment effect by calculating the change in SBP, DBP, K, eGFR, ARC, and SF-36 scores before and after each treatment. Comparisons were made between patients with uPA and bPA during MRA treatment (period A–B vs period C–D in **Figure 1**), between MRA in patients with bPA and ADX in those with uPA (period A–B vs period C–E in **Figure 1**), and between MRA and ADX in patients with uPA (period C–D vs period C–E in **Figure 1**). In patients with uPA, ADX significantly reduced SBP and eGFR compared to MRA. The increase in K observed with MRA treatment was greater in patients with uPA than in patients with bPA. In patients with uPA, ADX led to a significantly greater increase in K compared to MRA (**Figure 3**). Regarding SF-36 scores, no significant differences were observed in changes across domains between patients with uPA and bPA during MRA treatment. On the other hand, in patients with uPA, ADX led to significantly greater improvements in VT compared to MRA in patients with bPA (1.4 ± 8.8 vs 4.9 ± 5.8; *P <*0.05) (**Figure 2C**).

## Discussion

### Efficacy of MRA and ADX treatments

This study compared the efficacy of MRA and ADX treatments in patients with uPA. Few studies have collected data on longitudinally assessedpatients with uPA and compared the effectiveness of MRA treatment and ADX, and this is the first study to specifically examine changes in QOL over time. When comparing the effects of MRA treatment between patients with bPA and uPA, no significant differences were observed in the changes in clinical and biochemical parameters, except for a greater increase in K (**Figure 3**). Among patients with uPA, MRA treatment significantly improved SBP and K relative to pre-treatment levels, whereas ADX led to even greater improvements in these parameters (**Table 1**). In terms of QOL, SF-36 scores showed no significant improvement with MRA treatment in patients with uPA, while ADX significantly enhanced scores compared to pre-treatment levels (**Figure 2B**). These findings suggest that while MRA is effective for treating uPA, ADX provides superior outcomes, particularly in improving SBP, K levels, and QOL. In particular, the lack of improvement in QOL with MRA treatment could strengthen the indication for ADX in patients with uPA.

### Relevance of the assessment period

In this study, the median duration of MRA treatment was 85 days (IQR: 53–92), approximately equivalent to the 3 months follow-up period after ADX treatment. However, the increase in ARC remained insufficient following both MRA and ADX treatments. Hundemer et al. (19) reported that patients achieving a PRA of ≥ 1 ng/mL/h through MRA treatment experienced an incidence of atrial fibrillation comparable to that observed post-ADX. Further, Hundemer et al. (34) demonstrated that the incidence of cardiovascular events in PA patients achieving a PRA of ≥ 1 ng/mL/h with MRA treatment improved to levels comparable to those observed in essential hypertension. Similarly, Yoshida et al. (35) found that patients achieving an ARC of ≥ 5 pg/mL through MRA treatment for PA showed improved salt sensitivity.

These findings highlight the importance of elevating ARC through treatment. However, in our study, the median ARC of ≥ 5 pg/mL (equivalent to a PRA of ≥ 1 ng/mL/h (6)) was not attained, even after ADX treatment (**Table 1**). This could be attributed to the relatively short treatment duration and the pronounced impacts of excess aldosterone associated with uPA. Previous studies analyzing PRA at 6 months and between 6 and 12 months after ADX in patients with uPA consistently reported insufficient improvement in PRA levels during either period (21, 36). In contrast, a previous study analyzing PRA 12 months after ADX or MRA treatment in patients with uPA reported an improvement in PRA levels to >1 ng/mL/h (24). These findings suggest the need for longer-term observation, such as 12 months or more, to achieve optimal outcomes. In addition, in the present study, the median values of blood pressure and K levels became in normal range following MRA treatment in both patients with bPA and uPA, which may have led to a situation in which the MRA dose was not further increased before sufficient improvement in the suppressed renin levels was achieved. It is important to conduct a study comparing the therapeutic effects after adjusting the dosage of MRAs with the aim of improving renin suppression.

### MRAs selected for treatment of PA patients

In this study, spironolactone, eplerenone, or esaxerenone was used as MRA treatment. Esaxerenone is a new MRAs without a steroid backbone. In a study in rats, esaxerenone was reported to have a longer duration of effect, to reduce the urinary Na^+^/K^+^ ratio and to be more selective for MR, with no effect on glucocorticoid, androgen, and progesterone receptors as compared to spironolactone and eplerenone (37). In a phase III clinical trial, Satoh et al. (30) reported that treatment with esaxerenone significantly reduced SBP by 17.7 mmHg [95% confidence interval -20.6, -14.7] and DBP by 9.5 mmHg [-11.7. -7.3] in patients with PA. In our previous report (18), esaxerenone significantly reduced SBP, DBP, urinary albumin excretion, and N-terminal pro-brain natriuretic peptide, increased ARC, and improved QOL in patients with PA. Thus, esaxerenone is an MRA that has been shown to be effective in patients with PA, and it is reasonable that it was used in this study.

### Comparison with previous reports

Satoh et al. (38) conducted a systematic review investigating differences in treatment efficacy between MRAs and ADX. While their findings indicated that ADX is typically used for uPA and MRAs for bPA, no studies directly compared these treatments for the same condition. Several other studies have examined MRA and ADX treatments (19, 21–25, 36). Notably, Katabami et al. (21) and Wu et al. (24) used extensive national case registries to compare the efficacy of MRAs and ADX in patients with uPA. Katabami et al. focused on blood pressure and K as treatment outcomes, while Wu et al. evaluated mortality and major adverse cardiovascular events. Both studies concluded that ADX was superior to MRAs. The rationale for employing MRA treatment in patients with uPA in these registry-based reports is unclear, and patient selection bias may have influenced these findings.

In a related retrospective analysis, Zhang et al. (25) found that preoperative MRA treatment in patients with uPA effectively controlled perioperative blood pressure and reduced the incidence of postoperative hyperkalemia compared to groups not receiving MRA. While this study is similar to the current one in observing metrics such as blood pressure and K over time, it differs in its primary focus, which did not include QOL. Velema et al. (14) and Murck et al. (27) evaluated QOL before and after MRA and ADX treatments but targeted different pathologies, with patients with uPA undergoing ADX and patients with bPA receiving MRAs.

The present study is unique in that it aimed to mitigate treatment selection bias by directly comparing metrics such as blood pressure, hormone levels, and QOL in the same patients with uPA undergoing both MRA and subsequent ADX treatments.

### eGFR reduction

In this study, eGFR decreased significantly in patients with uPA following both MRA and ADX treatments. This reduction is likely due to decreased aldosterone-mediated intraglomerular hyperfiltration (39). ADX treatment led to a greater eGFR reduction compared to MRA, indicating greater improvement in intraglomerular pressure. While both treatments appear to reduce eGFR primarily through hemodynamic changes, often referred to as the “initial dip.” A slower rate of long-term eGFR decline might be expected if urine albumin/protein excretion is reduced; however, we did not assess this and further study is warranted.

### QOL

Previous studies have shown that patients with PA often experience reduced QOL, with treatments such as MRA or ADX improving QOL in certain populations (9–18). Consistent with other studies utilizing the SF-36 questionnaire (10–14, 16–18), this study also compared QOL data before and after MRA treatment in patients with uPA and bPA, observing changes over time. The patients with uPA in this study exhibited symptoms such as hypokalemia and refractory hypertension, and their initial SF-36 scores were significantly lower than those of patients with bPA (**Figures 2A, B**). Although the mechanisms underlying the reduced QOL in patients with uPA are not completely understood, studies on rats showed that aldosterone administration leads to an increase in anxious behavior (40), whereas eplerenone administration reduces anxiety (41). These findings suggest that mineralocorticoid receptor overactivation may contribute to anxiety-related symptoms. Furthermore, PA is frequently associated with obstructive sleep apnea syndrome (OSAS), which may negatively affect the physical aspects of QOL (42). Notably, both MRA and ADX treatments have been reported to alleviate OSAS symptoms (43, 44).

In our study, SF-36 scores improved significantly in patients with bPA after MRA treatment but not in patients with uPA. Scores remained significantly lower in patients with uPA compared to patients with bPA. However, following ADX, SF-36 scores in patients with uPA improved to levels that were comparable to those in patients with bPA (**Figures 2A, B**). When examining the changes in SF-36 scores, no significant differences were observed between patients with uPA and bPA after MRA treatment. In contrast, improvements in SF-36 scores were significantly greater after ADX in patients with uPA than after MRA in patients with bPA (**Figure 2C**). These results may reflect the greater degree of deterioration in QOL in patients with uPA compared to patients with bPA. Before MRA treatment, patients with uPA exhibited significantly lower SF-36 scores across all domains except PF, BP, SF compared to patients with bPA, even though control of their blood pressure was comparable to that of patients with bPA (**Table 1**). During the same duration of MRA treatment, patients with uPA may not achieve the same absolute level of improvement in QOL as patients with bPA, even if the magnitude of the change in SF-36 scores is similar. Biochemically, K and ARC levels were significantly lower in patients with uPA compared to those in patients with bPA before treatment. Although these levels improved following MRA treatment, they remained significantly lower than those in patients with bPA (**Table 1**). In the present study, the maximum dose of MRA could not administered to many patients, due to the relatively short duration of treatment (3 months) before surgery and a reduction in eGFR due to reduced aldosterone-mediated intraglomerular hyperfiltration. Even after MRA treatment, patients with uPA still had significantly lower ARC than patients with bPA, suggesting that MR activity may not have been sufficiently suppressed in these patients. The results may have differed if the dose of MRA had been increased to the maximum, but doing so within the limited treatment period due to the reasons mentioned above. Consequently, comparisons between patients with bPA and uPA during MRA treatment should be interpreted with caution.

### Limitations

Our study has several limitations. First, the small number of patients limits the generalizability of our findings and reduces the ability to draw firm conclusions. Larger studies involving patients with uPA are required to provide more robust findings. Second, the same patients were sequentially treated with MRAs followed by ADX, and outcomes were assessed after a relatively short period (approximately 3 months). This approach complicated direct comparisons of treatment efficacy as the effects of the two treatments were difficult to distinguish. Therefore, it is not possible to conclude a cause-and-effect relationship from the result of the present study. Future studies with longer follow-up periods and alternative study designs that match patient backgrounds, such as propensity score matching, may better clarify these differences. Third, in patients with uPA, MRAs were administered for a short-term period prior to ADX to stabilize blood pressure and K levels, whereas in patients with bPA, they were administered with the aim of achieving long-term and sustained improvement of clinical and biochemical outcomes. Therefore, the therapeutic effects of MRAs in bPA and uPA should be carefully considered, and the present study does not allow for a definitive conclusion regarding the differences in MRA efficacy between bPA and uPA. Fourth, the patients were instructed to change their lifestyle, such as restricting salt intake, and to take oral medication. However, it is not known exactly whether the patient actually followed the instructions, so the possibility that lifestyle influences the results cannot be ruled out. Fifth, three types of MRAs were used in the present study. These differ in their selectivity for MR, and may have different therapeutic effects. Therefore, it is desirable for future research to be conducted using a standardized type of MRAs. Finally, in the present study, the minimal clinically important difference (MCID) of SF-36 scores could not have been shown. MCID is important to assess the degree of improvement in an index that is perceptible to the patients. Therefore, careful consideration is needed to determine whether the degree of improvement in SF-36 scores in the result of the present study represents a clinically meaningful improvement in QOL.

## Conclusions

This study demonstrated that ADX treatment was more effective than MRA treatment in improving SBP and K levels in patients with uPA in a short-term analysis. QOL, as measured by the SF-36, was significantly lower in patients with uPA compared to patients with bPA before treatment. While MRA treatment did not lead to significant improvements in QOL, ADX treatment elevated QOL scores to levels comparable to those observed in patients with bPA.

## Data Availability

Some or all datasets generated during and/or analyzed during the current study are not publicly available but are available from the corresponding author on reasonable request.
